# Lung volume segmentation in fetal MRI: super-resolution reconstructions improve inter-rater reliability

**DOI:** 10.1186/s41747-025-00628-4

**Published:** 2025-09-09

**Authors:** Kelly Payette, Julia Geiger, Michael Zellner, Céline Steger, Christian J. Kellenberger, Ruth Tuura, Raimund Kottke, Andras Jakab

**Affiliations:** 1 Center for MR-Research, University Children’s Hospital Zurich, University of Zurich, Zurich, Switzerland; 2 Neuroscience Center Zurich, University of Zurich, Zurich, Switzerland; 3 Department of Early Life Imaging, School of Biomedical Engineering and Imaging Sciences, King’s College London, London, UK; 4 Department of Diagnostic Imaging and Intervention, University Children’s Hospital Zurich, University of Zurich, Zurich, Switzerland; 5 University of Zurich, Zurich, Switzerland; 6 Children’s Research Center, University Children’s Hospital Zurich, University of Zurich, Zurich, Switzerland; 7 Pediatric Cardiology, Pediatric Heart Center, Department of Surgery, University Children’s Hospital Zurich, University of Zurich, Zurich, Switzerland

**Keywords:** Image processing (computer-assisted), Lung volume measurements, Magnetic resonance imaging, Pregnancy, Reproducibility of results

## Abstract

**Background:**

Fetal MRI is increasingly used to investigate fetal lung pathologies, and super-resolution (SR) algorithms could be a powerful clinical tool for this assessment. Our goal was to investigate whether SR reconstructions result in an improved agreement in lung volume measurements determined by different raters, also known as inter-rater reliability.

**Materials and methods:**

In this single-center retrospective study, fetal lung volumes calculated from both SR reconstructions and the original images were analyzed. Three radiologists manually segmented the fetal lungs and rated the image quality of all images and reconstructions. Fetal lung volumes were calculated, and the coefficient of variation (CV) was determined for each set of images. Bland–Altman plots were generated, and intraclass correlation coefficients (ICCs) were calculated. A one-sided paired Wilcoxon test was used to compare the fetal lung volume CVs, and a two-sided paired *t*-test was used to compare the lung volumes. The quality ratings were compared using a two-sided paired Wilcoxon test.

**Results:**

A total of 98 fetal scans with gestational ages from 19 to 37 weeks were evaluated. There was a significantly lower CV in the lung volumes segmented from the SR reconstructions (*p* < 0.001), and the ICCs of the reconstructions were higher than those determined from the original images. Bland–Altman plots demonstrated better agreement in the SR reconstruction lung volumes. No significant differences in quality ratings or lung volumes were found.

**Conclusion:**

SR reconstructions of the fetal lungs in MRI enabled better inter-rater reliability of fetal lung volume assessment.

**Relevance statement:**

SR reconstructions of the fetal body, obtained through fetal MRI, can be a valuable tool for improving the inter-rater reliability of fetal lung volume measurements, a crucial clinical biomarker for assessing fetal development and predicting pregnancy outcomes.

**Key Points:**

- Deformable slice-to-volume reconstructions of fetal body MRI could be a valuable clinical tool.
- Quantitative advantages of fetal body MRI reconstructions need to be proven.
- Fetal body MRI reconstructions improved inter-rater reliability in lung volume measurements.

**Graphical Abstract:**

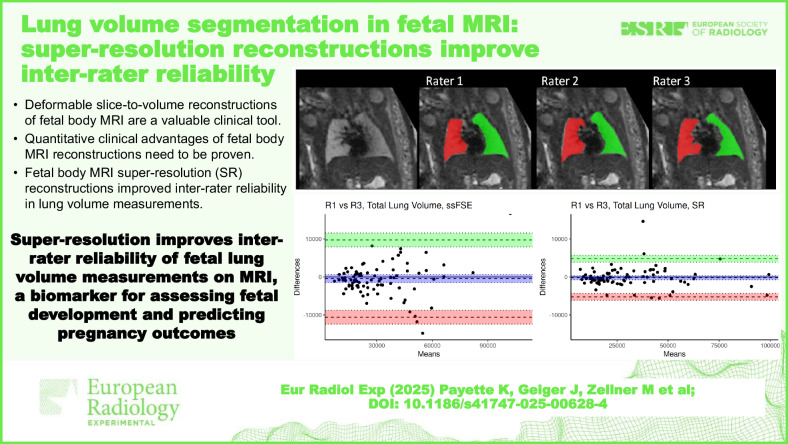

## Background

Fetal magnetic resonance imaging (MRI) is an important complementary tool to ultrasound in prenatal care. While fetal MRI is primarily utilized for assessing and diagnosing central nervous system pathologies, its usefulness has also been shown for the assessment, prenatal diagnosis, and perinatal treatment planning for multiple non-central nervous system indications [1–4]. Fetal MRI lung volumetry is an important prognosticator for several pathologies, including lung hypoplasia due to congenital diaphragmatic hernia [5–8], intra-uterine growth restriction [9], giant omphalocele [10], and preterm birth [11]. Total fetal lung volume reference curves from MRI have been established across a wide range of gestational ages (GAs) for normal fetuses [12–14]. As fetal lung volumetry based on MRI is increasingly used for prognostication in clinical practice (for a detailed review on fetal lung assessment in utero, see [15]), it is of the utmost importance to obtain accurate and reliable fetal lung volume measurements.

The acquisition of high-quality fetal MRI is challenging due to the presence of unpredictable and uncontrollable fetal motion. This has been addressed through the use of fast two-dimensional sequences such as single-shot fast spin-echo (ssFSE) or half-Fourier single-shot turbo spin-echo, which produce anisotropic images with excellent in-plane resolution (0.5–1 mm^2^) and thick slices (3–5 mm). Movement is often present between the slices, and it can be very challenging to acquire images in the standard diagnostic planes, resulting in off-plane images. Alternatively, time can be invested in acquiring in-plane images, leading to clinical workflow delays.

**Fig. 1:**
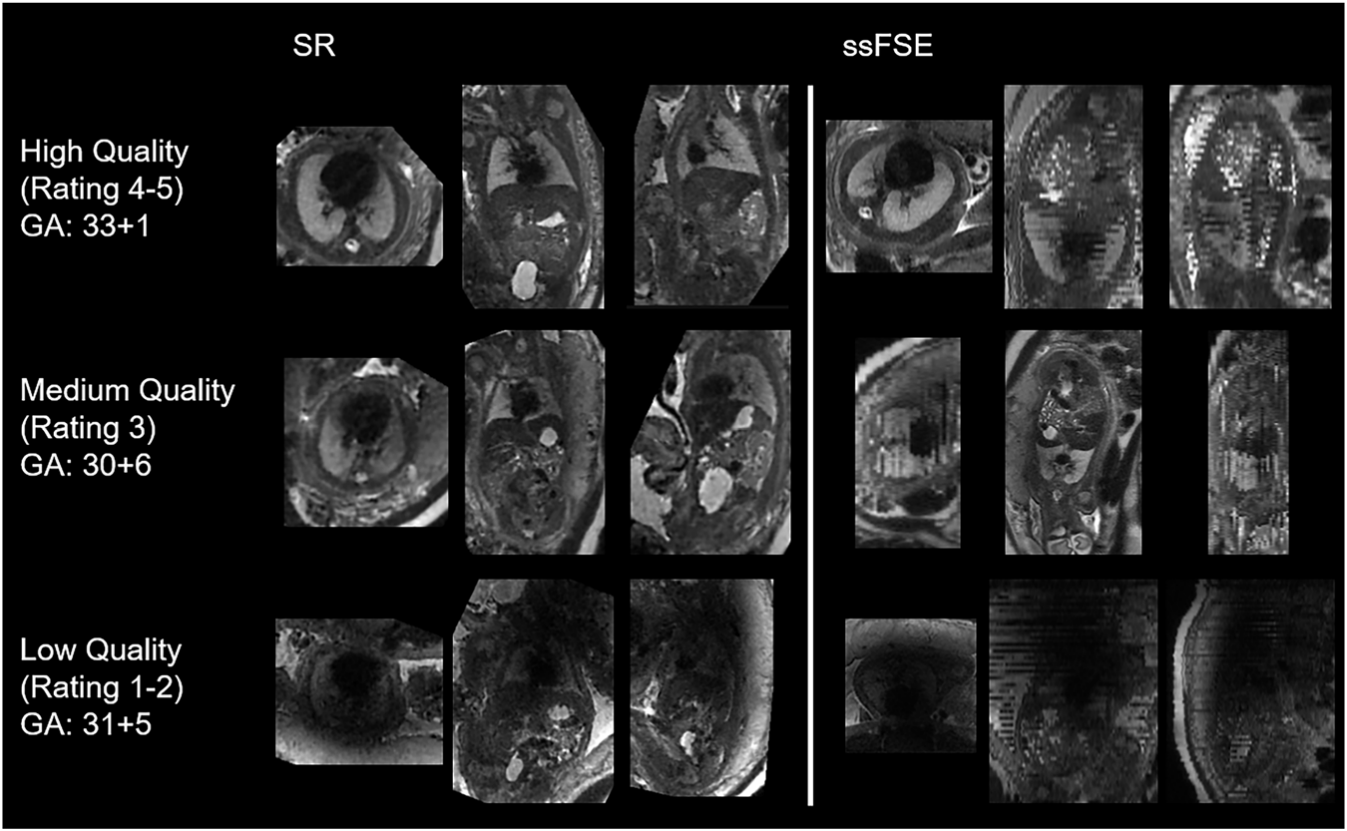
Examples of the SR reconstructions and the original ssFSE images. A low-quality case (rating 1 or 2), a medium-quality case (rating 3), and a high-quality case (rating 4 or 5) are shown for each. The in-plane image of the ssFSE image demonstrates excellent resolution, whereas off-plane, thick slices with inter-slice motion can be observed. The low-quality example is further impacted by signal dropout and motion. SR, Super-resolution; ssFSE, Single-shot fast spin-echo

Recently, it has become possible to reconstruct super-resolution (SR) reconstructions of the fetal body from multiple clinically acquired scans with a low off-plane resolution using an SR method called deformable slice-to-volume reconstruction (Fig. 1). It takes multiple non-isotropic ssFSE scans as input and reconstructs a single, high-resolution volume as output, applying motion correction and outlier rejection steps [16, 17]. While an increasing number of studies opt to use fetal body reconstructions for analysis of fetal lung volumes [11, 18–20], the majority of studies continue to utilize the acquired ssFSE images to determine fetal lung volumes [5, 12, 13, 21, 22], despite the growing use of SR reconstructions and the literature hypothesizing that SR reconstructions of the fetal lung may provide a more accurate evaluation of overall lung size [15].

This study aims to investigate the potential clinical benefit of using SR reconstructions in lung volumetry calculations. Here, we aim to determine whether fetal lung volumes calculated from SR reconstructions are equivalent to those calculated by the original acquired scans, and to investigate the inter-rater reliability of those fetal lung volume calculations in both the SR reconstructions and the original acquired scans. We retrospectively analyze fetal MRI scans and compare the volume and inter-rater reliability of fetal lung volumes calculated from segmentations done by three radiologists in both the original images and SR reconstructions. Lung segmentations and volumes are calculated with fetuses across a wide range of GAs, and the quality of the acquired fetal MRI scans is investigated.

## Materials and methods

### Patients

Pregnant women were scanned as part of their routine clinical care and gave informed written consent for the re-use of their data for research purposes. The images were acquired at the University Children’s Hospital Zurich between September 2019 and July 2020. The ethical committee of the Canton of Zurich, Switzerland (KEK) approved the retrospective studies that collect and analyze fetal MRI data (decision numbers: 2016-01019 and 2022-0115). Fetuses with a lung pathology were excluded from this analysis.

### MRI protocol

Fetal MRI was acquired using 1.5-T or 3-T whole-body scanners (Signa Discovery MR450 or MR750, GE Healthcare, Waukesha, WI, USA). For each case, multiple T2-weighted ssFSE sequences were acquired using either an 8-channel cardiac coil or a body coil, depending on the scanner. The imaging plane was oriented relative to the fetus, and at least one axial, sagittal, and coronal scan was acquired. Each ssFSE sequence had an in-plane isotropic resolution of 0.5–0.8 × 0.5 × 0.8 mm^3^, a slice thickness of 2.5–5 mm, and an acquisition time from 1:00 to 1:30 min:s. The sequence parameters were the following: repetition time 2,000−3,500 ms; echo time ≥ 120 ms; flip angle 90°; sampling percentage 55%. The field of view (ranging 200–240 mm) and image matrix (256 × 224 at 1.5 T; 320 × 224 at 3 T) were adjusted depending on the GA and size of the fetus, as the FOV and image matrix may need to be increased depending on the size of the fetus, especially at later GAs. The total scan time lasted 50–60 min.

For each examination, the technician and radiologist acquired as many sequences as they deemed necessary according to their clinical experience, meaning no consistent number of scans was acquired. The ssFSE scans were manually reviewed for each examination, and a subset of the highest quality scans (at least one in each orientation) was selected, resulting in a range of 4–13 scans. The deformable slice-to-volume reconstruction was run using these scans as input to obtain an SR reconstruction with a resolution of 0.8 × 0.8 × 0.8 mm^3^ [16]. Each reconstruction took 10–30 min on an Intel(R) Xeon(R) Gold 6130 CPU with 64 cores.

### Image analysis

**Fig. 2:**
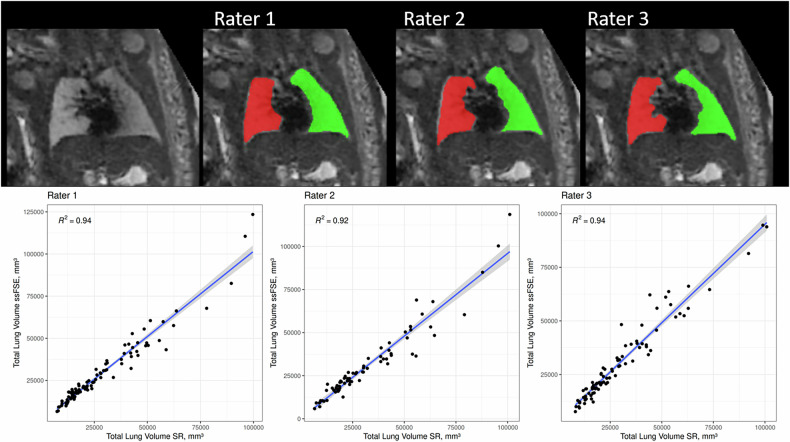
Top: fetal lung segmentations (GA 33.1 weeks) performed by each radiologist on a SR reconstruction lung segmentation (red, right lung; green, left lung); Bottom: Intra-rater agreement between the ssFSE fetal lung volumes and the SR fetal lung volumes for each rater. *R*^*2*^ values were calculated to be 0.94, 0.92, and 0.94 for Rater 1, Rater 2, and Rater 3, respectively. SR, Super-resolution; ssFSE, Single-shot fast spin-echo

Three board-certified radiologists with fetal MRI experience ranging from 1 year to 10 years segmented the left and right fetal lungs in both the SR volume and in an ssFSE scan. Each radiologist rated the quality of each image (ssFSE and SR) using a Likert scale from 1 to 5, where a rating of 1 indicated poor quality and 5 indicated excellent quality. To best match a clinical scenario for fetal lung volume measurement, each radiologist was free to choose which ssFSE scan to segment in each subject, and they were able to decide if the scan was of sufficient quality to segment. The agreement in quality ratings was investigated using the Gwet coefficient [23, 24]. In the SR fetal lungs, every 4th–5th slice was manually annotated, and the FSL—FMRIB Software Library (version 6.0.0) and Insight Toolkit (ITK, version 5.3.0) morphological contour interpolation were used to create the final three-dimensional segmentation [25, 26]. In the ssFSE scan, every slice was manually segmented. All segmentations were performed with 3D Slicer [27, 28] (Fig. 2). The radiologists were blinded as to which set of ssFSE scans matched each reconstruction, and did not have access to the MRI reports and patient information (*i.e.*, GA, MRI indication, findings).

### Statistical analysis

The interobserver reliability of the segmentations was analyzed by looking at the difference in coefficient of variation (CV) and intraclass correlation coefficient (ICC) of fetal lung volumes (left lung, right lung, both lungs) determined from the segmentations performed by each of the three radiologists. A two-sided paired *t*-test was performed after checking for normality to determine if the lung volumes from the SR reconstructions were different than those determined from the ssFSE images, and a paired, two-sided Wilcoxon test was used to determine if the quality ratings of the ssFSE images were different than those of the SR reconstructions.

Intra-rater agreement of the lung volumes was determined by calculating a line of best fit of the SR reconstruction lung volumes *versus* the ssFSE lung volumes, and calculating the *R*^*2*^. A one-sided paired Wilcoxon test was performed to determine if the CV in the fetal lung volumes determined from the SR reconstructions was lower than the CV in the fetal lung volumes determined from the ssFSE images. ssFSE scan segmentations and SR reconstruction segmentations were included in the CV calculation if at least two radiologists performed the segmentations. Next, Bland–Altman plots were created to visualize the agreement between each of the three observers. Finally, a linear analysis of variance of the CV was performed on the data from the ssFSE images and the SR reconstructions to look at the impact of GA in weeks, median image quality rating, and magnet strength (1.5 T/3 T). An analysis of variance was performed to look at the relationship between median image quality rating and GA. All analysis was performed with R v4.0.3 [29], and *p*-values less than 0.05 were considered to be significant.

## Results

### Patients included

Clinical fetal MRI with written research consent was performed in 118 pregnant women during the time frame. Thirteen cases were excluded due to multiple pregnancies (twins/triplets), and 7 cases were excluded due to the presence of pulmonary pathology, resulting in 98 cases included in this study, with GA ranging from 19 weeks to 37 weeks (26.2 ± 4.2 weeks, mean ± standard deviation). We included 19 subjects who were scanned twice throughout their pregnancy. The repeated scans were performed at different times of the gestation as part of the follow-up of the clinical condition. The raters were blinded to this information, and therefore, the corresponding lung volume measurements were treated as independent observations. The study subject characteristics are summarized in Table 1.

**Table 1:** Subject characteristics (*n* = 98)

Variables	Details
GA (weeks, mean ± standard deviation)	26.2 ± 4.2
Field strength	1.5 T, 42 examinations 3 T, 56 examinations
Abnormal fetuses (*n* = 91)	47 spinal dysraphisms 34 other neuro pathologies 5 body non-lung pathologies 4 head/neck pathologies 1 oligohydramnion
Normal fetuses (*n* = 7)	Based on radiological reports

Pregnant women were scanned as part of their routine clinical care, and a ‘normal’ classification resulted after abnormal findings in the lungs were ruled out by MRI

### Manual segmentation

All three radiologists reviewed all ssFSE images and SR reconstructions for the 98 cases and provided quality ratings. The number of scans segmented by each radiologist is shown in Table 2, as each radiologist independently determined if the scan was of a sufficient quality to be segmented. There were 82 of 98 ssFSE cases and 79 of 98 SR cases where all three radiologists provided a segmentation. There were 67 of 98 cases where all radiologists provided both an ssFSE and SR segmentation. Within the ssFSE cases where the radiologists were free to choose their own image to segment, all three radiologists chose the same scan to segment in 28 of the subjects. The percent agreement of the three radiologists, as calculated with the Gwet Coefficient of the ssFSE scans, was 0.89, and of the SR reconstructions was 0.90. There were some cases where the radiologist determined that the ssFSE scan could be segmented, but they were able to segment the SR reconstruction. Radiologist 1 was unable to segment 1 ssFSE case, and was not able to segment the SR reconstruction version of this case. Radiologist 2 was unable to segment 12 ssFSE cases, of which 8 had SR reconstruction segmentations. Radiologist 3 was unable to segment 7 ssFSE cases, of which 4 had SR reconstruction segmentations.

**Table 2:** Overview of the manual segmentations performed by the three radiologists (*n* = 98)

Radiologist	Number of ssFSE segmented	Number of SR segments	Quality ratings for ssFSE	Quality ratings for SR	Difference in quality, SR *versus* ssFSE (*p*-value)	Difference in lung volume, SR *versus* ssFSE (*p*-value)
1	97	91	2.81 ± 1.1	2.73 ± 1.2	0.709	0.117
2	86	79	2.37 ± 1.2	2.23 ± 1.2	0.693	0.069
3	91	88	2.58 ± 0.8	2.85 ± 1.3	0.054	0.133

Quality ratings are given as mean ± standard deviation

*SR* Super-resolution, *ssFSE* Single-shot fast spin-echo

There was no significant difference in the quality ratings between the SR reconstructions and the ssFSE scans for all radiologists (Table 2). For 23 SR reconstructions and 19 ssFSE cases, all three radiologists reported the same quality rating.

### Fetal lung volumetric analysis

**Fig. 3:**
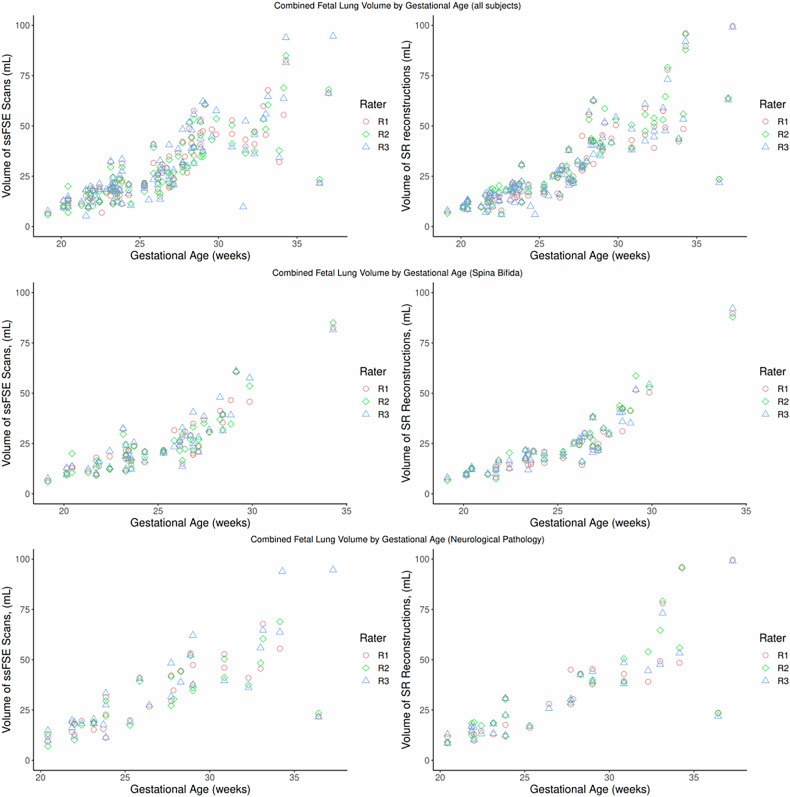
Combined fetal lung volume (left and right lungs) of each case *versus* GA in ssFSE scans (left) and SR reconstructions (right) of the three observers. The top row displays all subjects, the middle row displays the cases with spina bifida, and the bottom row shows the cases with a neurological pathology (excluding spina bifida cases). The minor decrease in volumes observed at 30–32 gestational weeks, seen in the top row (all data) is not as pronounced when the data is separated into subgroups. SR, Super-resolution; ssFSE, Single-shot fast spin-echo

From the lung segmentations, total fetal lung volume for each case was calculated (Fig. 3). Due to the large number of spina bifida cases in the cohort (*n* = 47), they have been separated out of the subsection ‘Neurological Pathology’. The lung volumes of subjects fell within the normal ranges for their GA according to the literature [12–14].

There was no overall difference between the combined volumes of the left and right lungs determined by the ssFSE images *versus* the volumes determined by the SR reconstructions (Table 2). There was also high intra-rater agreement of fetal lung volumes between the ssFSE images and SR reconstructions. When the fetal lung volumes determined from the ssFSE images were compared to the fetal lung volumes from the SR reconstructions, *R*^*2*^ values of 0.94, 0.92, and 0.94 were calculated for the individual radiologists (Fig. 2).

The mean CV and ICC of the left lung, right lung, and both lungs combined can be found in Table 3. As hypothesized, the interobserver variability was significantly lower in the SR recons in comparison to the ssFSE scans: 5.69 ± 4.87 (mean ± standard deviation) *versus* 7.8 5 ± 5.60, respectively, *p* < 0.001). When taking the left and right lung volume measurements as independent observations, the inter-observer variability of lung volume measurements was still significantly lower in the SR reconstructions (right, *p* < 0.001; left, *p* = 0.043). As mentioned in the ‘Manual segmentation’ section, all three radiologists chose the same ssFSE scan to segment in 28 of the cases. A one-sided paired Wilcoxon test was performed on this subset of 28 cases. Here, there was no significant difference between the SR and ssFSE CV. The ICC was also calculated between the three observers, and in all cases, the ICC of the fetal lung volumes segmented from the SR reconstructions (0.995–0.996) was higher than the ICC of the fetal lung volumes segmented from the ssFSE images (0.987–0.989, Table 3).

**Table 3:** Inter-rater reliability analysis

Lung side	CV for ssFSE	CV for SR	Difference in CV, SR *versus* ssFSE (*p*-value), all cases, (*n* = 98)	*p*-value (difference in CV between ssFSE and SR, same ssFSE), *N* = 28	ICC for ssFSE	ICC for SR
Left	8.86 ± 6.07	7.40 ± 5.41	0.034	0.987	0.989	0.995
Right	8.41 ± 6.71	5.97 ± 5.19	< 0.001	0.787	0.987	0.996
Left and right	7.85 ± 5.60	5.69 ± 4.87	< 0.001	0.772	0.989	0.996

To determine if the CV of the SR lung volumes was lower than the CV of the ssFSE lung volumes, a paired, one-sided Wilcoxon test was used, as well as the ICC values of the determined lung volumes by each rater (ICC values greater than 0.90 are indicative of excellent reliability, according to ref. [43]). Same ssFSE refers to the cases where all three radiologists chose the same ssFSE image to segment. CV data are given as mean ± standard deviation

*CV* Coefficient of variation, *ICC* Intraclass correlation coefficient, *SR* Super-resolution, *ssFSE* Single-shot fast spin-echo

**Fig. 4:**
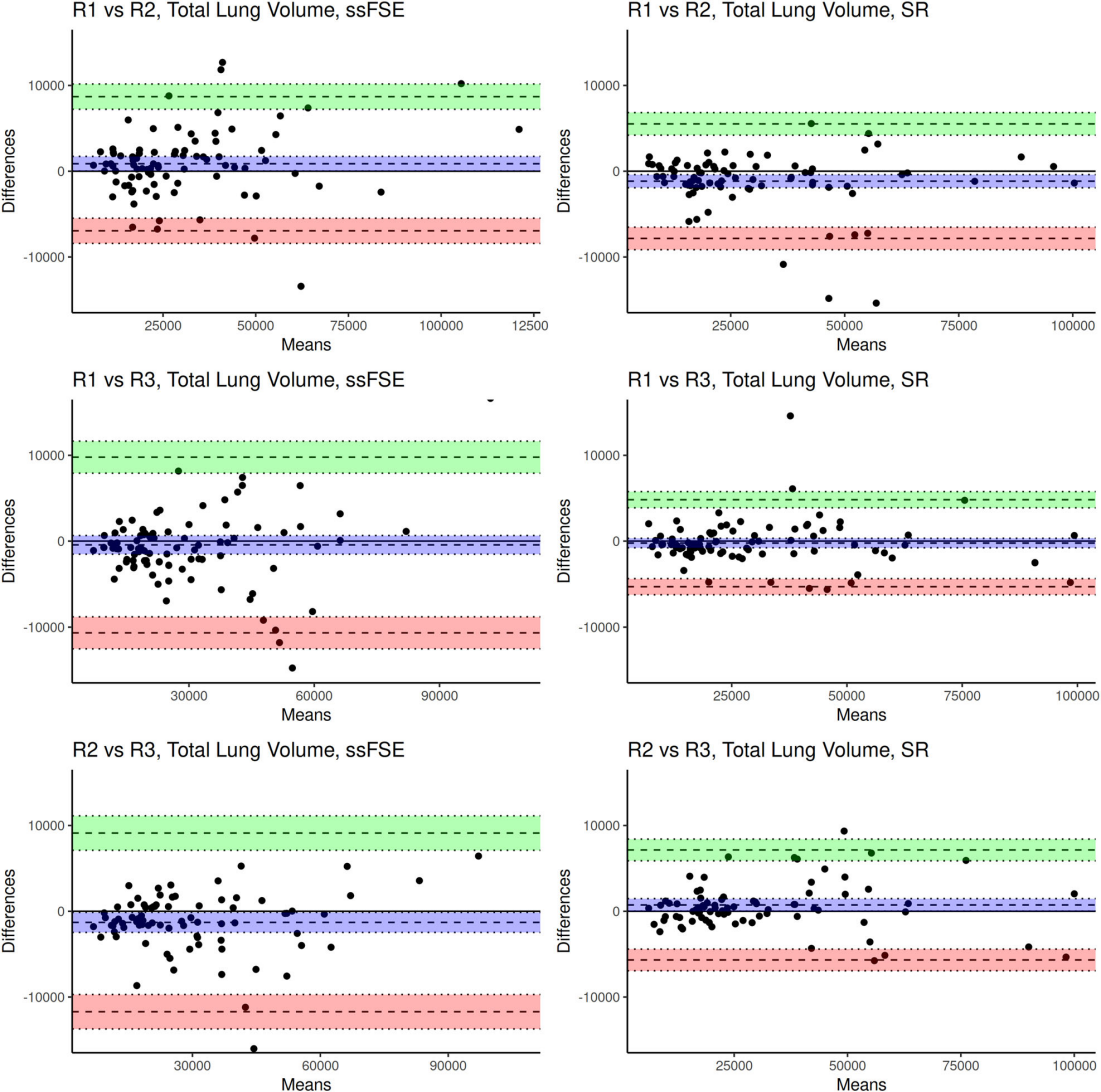
Bland–Altman plots showing the inter-observer agreement for fetal lung segmentation in ssFSE images and SR reconstructions. The dotted lines indicate the 25th and 75th quartiles; the shaded regions indicate the confidence interval. R1, R2, and R3: Raters 1, 2, and 3. The *x*-axis shows the mean total lung volume (mL) between the two raters; the *y*-axis displays the difference in total lung volume (mL) between the two measurements. The spread between the upper and lower dotted lines for each comparison (ssFSE, SR) is as follows: R1 *versus* R2: 15724, 13414; R2 *versus* R3: 20515, 12800; R1 *versus* R3: 20318, 10037. In each comparison, the spread between the upper and lower dotted lines is smaller in the SR reconstruction comparisons than in the ssFSE comparisons. SR, Super-resolution; ssFSE, Single-shot fast spin-echo

Figure 4 shows the Bland–Altman plots of the agreement between each observer. Visually, the differences between the radiologists’ segmentations are consistently smaller in the SR reconstructions. When looking at the difference between the upper and lower dotted lines (corresponding to the 25th and 75th percentiles), it is smaller in the SR reconstruction comparisons than in the ssFSE comparisons for each rater comparison.

The impact of GA, magnet strength, and image quality on the CV was explored, and an analysis of variance was performed. Both GA and magnet strength were found to have no impact on the CVs determined from both the ssFSE (*p*-values 0.450 and 0.819, respectively) and SR reconstructions (*p*-values 0.343 and 0.085, respectively). CVs were impacted by the image quality in both the ssFSE images (*p*-value 0.005), but not by the SR reconstructions (*p*-value 0.057). No relationship between median image quality and GA was found in the ssFSE images or the SR reconstructions (*p*-values 0.179 and 0.578, respectively).

## Discussion

We compared the inter-rater reliability of fetal lung volume measurements segmented from clinically acquired ssFSE scans to those obtained using SR reconstructions. The SR reconstructions resulted in more reliable fetal lung segmentations and volume estimates than the original ssFSE scans. We found a high intra-rater agreement between the lung volume measurements based on ssFSE and SR reconstructions for each radiologist. While some of the SR reconstructions were not segmented due to low quality (6 by R1, 19 by R2, and 10 by R3), the segmentations of the remaining SR reconstructions were more reliable, demonstrating more agreement between the three observers in both the CV and ICC measurements. In some cases, the radiologist determined that the original ssFSE images were of too low quality to segment, but the SR reconstructions were able to be segmented (0 of 1 for R1, 8 of 12 for R2, and 4 of 7 for R3), indicating that SR reconstructions of the fetal body may indeed help to increase the number of usable scans on the individual case level, even if the group quality rating was not significantly different.

A comparison of the CV in the 28 cases where the radiologists chose the same ssFSE scan to segment found no significant differences between the ssFSE and SR reconstruction segmentations, indicating that one of the main benefits of SR reconstructions is that it provides the same image for individuals to segment, in agreement with the literature [30]. There may be inherent differences in volumes determined from different ssFSE scans, especially as the borders of the lung may be obscured in the thick slices present in ssFSE scans. This is particularly important as several repetitions are typically acquired clinically due to fetal motion or the incorrect positioning of the imaging plane based on a previous scan, and raters may choose different ssFSE images based on varying criteria, leading to less reliable lung volumetry.

The improvement in reliability from the SR reconstructions may also arise from the motion correction of the images and orientation to a standard imaging plane. Previous work showed that deformable slice-to-volume reconstruction leads to superior correction of motion and fewer errors compared to other reconstruction techniques [16]. This type of reconstruction is particularly valuable for visualizing fetal organs, where motion can be nonrigid (that is, the body and fetal organs may experience deformations beyond rotation and translation). In cases where motion-induced artifacts hinder the visibility of the fetal lung on the first or last ssFSE slices, a considerable bias can emerge if these slices are not included in the volumetric measurements. Interestingly, there was no significant difference in the quality ratings given by the radiologists to the SR reconstructions and the ssFSE images. The quality of the reconstructed volume is not independent of the quality of the ssFSE images. In order to create clinically usable reconstructions, ssFSE images of a decent quality and number are required to create usable reconstructions, as not all reconstructions were of sufficient quality to allow segmentation. In addition, the images were not acquired with the intention of performing SR reconstruction. In order to increase the probability of a high-quality SR reconstruction, multiple sequences, typically two to three, in each orientation need to be acquired to ensure adequate input information for the SR algorithm. As a result, some cases did not have enough ssFSE images acquired for an optimal reconstruction. There was a weak relationship between the CV and the image quality of SR reconstructions, and a significant relationship between the CV and the image quality of the ssFSE images. This relationship was expected, as higher quality images tend to have clearer borders, making them easier to segment. There has been a recent interest in automated quality control systems for fetal brain MRI to aid in optimal image acquisition [31, 32]. Real-time quality control feedback at the scanner could result in higher quality images acquired, as the individual scanning would have a quantitative understanding of the quality of the images already acquired, and if they need to perform further acquisitions. Current tools being developed are for the fetal brain, but tools for fetal body imaging could be just as helpful for reliable, high-quality fetal image acquisition.

GA seemed not to play a role in the variability of fetal lung segmentations, which was unexpected. Higher GA (over 30 weeks) tends to have better image quality due to decreased fetal motion, as the fetus is larger. We expected the older fetuses to be more reliably segmented, but this was not the case. In addition, no relationship between magnet strength and CV was found, meaning this technique can be used on both 1.5-T and 3-T magnets with the same reliability.

Despite the slight decrease in lung volumes of fetuses scanned between gestational weeks 30 and 32 (Fig. 3), the lung volumes still fall within the normal range [12, 13]. This is most likely due to the various pathologies that were scanned and the unequal number of fetuses scanned at each GA. This trend is primarily found in fetuses with a neurological pathology (Fig. 3, bottom row), but we cannot draw any conclusions regarding the lung volumes in neurological pathologies from this data. Davidson et al reported that while they found that the lung volumes calculated from the SR reconstructions were more reliable than those from the original images, the lung volumes were overall smaller [18]. We did not find this pattern of consistently lower lung volumes in the SR reconstructions, even though the same reconstruction algorithm was used, but this may be due to differences in the manual segmentation methodology used, or it could be influenced by the small sample size (*n* = 16) of the study by Davidson et al [18].

While it was not the main goal of our study to evaluate the clinical usability of SR, reconstructions may provide a clinical benefit in cases where a fetal lung volume measurement is required. In congenital diaphragmatic herniation, the protrusion of abdominal organs into the chest cavity compromises fetal lung development. Therefore, lung volume can reflect the compression of the affected lung and the development of the contralateral lung [33]. Fetal lung volume is clinically important, as it is associated with outcomes, such as the need for extracorporeal membrane oxygenation support [34, 35]. It is especially important to be able to reliably segment pathological fetal lungs, as pathological cases typically contain more anatomical variability, resulting in more challenging manual segmentations.

In the future, the clinical applicability of fetal body segmentations beyond the lung would be important to confirm, as many studies have shown that there are advantages to using fetal MRI for prenatal diagnosis and planning in fetal organs such as the liver [5, 36] and kidneys [37, 38]. The main limitation of our study is the heterogeneity of the clinical cohort and the lack of fetal lung pathologies. Therefore, we were not able to assess if SR reconstructions are more reliable than ssFSE images in fetuses with a lung pathology, where the anatomical configuration makes lung volumetry challenging. In order to obtain a comprehensive understanding of the practical use of this study in real clinical settings, future research should be performed on a fetal cohort of patients with lung anomalies. Recent studies have performed lung volume calculations of fetuses with CDH using the acquired low-resolution images rather than SR reconstructions [22, 39–41]. In each of these studies, determination of lung volume was found to play a role in outcome prediction, further highlighting the need for reliable fetal lung volume measurements beyond the pilot study performed by Davidson et al [18]. Additional limitations of the current study include the fact that we only looked at inter-rater reliability and not intra-rater reliability. Deep learning networks for the automatic segmentation of the normal fetal lung in SR reconstructions have been developed [20], but have not been used for pathologies. An automatic lung segmentation network for the acquired T2-weighted TSE images in CDH fetuses has been found to provide accurate segmentations [42], but it has not yet been demonstrated in SR reconstructions. In addition, the utility and reliability of SR reconstructions and automatic fetal lung segmentation networks should ideally be proven in a multicenter study.

SR reconstructions of the fetal lungs in MRI enable more reliable assessments of fetal lung volume compared to the original acquired low-resolution ssFSE images. However, high-quality ssFSE images are required to create usable reconstructions, as not all reconstructions can be segmented. In addition, the SR reconstructions should not be used to replace the ssFSE scans in diagnosis, but to supplement. While further work is needed to improve the image quality of the SR reconstructions such that all images can be segmented, SR reconstruction by deformable slice-to-volume reconstruction is a helpful tool that increases the number of usable scans and provides increased inter-rater reliability of fetal lung volume determination.

## Data Availability

The datasets generated and/or analyzed during the current study are not publicly available due to patient confidentiality reasons, but secondary data are available from the corresponding author upon reasonable request.

## Abbreviations

CV: Coefficient of variation
GA: Gestational age
ICC: Intraclass correlation coefficient
MRI: Magnetic resonance imaging
SR: Super-resolution
ssFSE: Single-shot fast spin-echo
